# ApoE and apoE receptors in brain lipid metabolism and AD

**DOI:** 10.1186/1750-1326-7-S1-L10

**Published:** 2012-02-07

**Authors:** Guojun Bu

**Affiliations:** 1 Department of Neuroscience, Mayo Clinic, Jacksonville, FL, 32224, USA

## Background

Brain lipids such as cholesterol play critical roles in neuronal membrane homeostasis and synaptic functions. Apolipoprotein E (apoE) is a major lipid transporter in the brain. Of the three human apoE isoforms (E2, E3 and E4), apoE4 is a strong risk factor for late-onset Alzheimer’s disease (AD). Brain apoE/lipoprotein particles, produced primarily by astrocytes, deliver cholesterol and other lipids to neurons to support synapses via apoE receptors, which belong to the low-density lipoprotein receptor (LDLR) family. To ultimately understand why apoE4 is a risk factor for AD, it is essential to study the differential functions of apoE isoforms in brain lipid transport and synaptic functions, and what specific roles apoE receptors play in these processes.

## Methods

We generated conditional knockout mice deleting a major apoE receptor LRP1 in forebrain neurons in adult mice. Effects of neuronal LRP1 deletion on brain apoE/lipoprotein metabolism, synaptic functions and memory performance were analyzed at different ages. The neuronal LRP1 deletion mice were further bred with APP/PS1 amyloid model mice to study the roles of LRP1 in brain Aβ metabolism.

## Results

We found that neuronal deletion of *Lrp1* impairs brain apoE and cholesterol metabolism. Consequently, the conditional *Lrp1* forebrain knockout (LRP1-KO) mice have decreased brain cholesterol, sulfatide and cerebroside; reduced dendritic spine density and branching; fewer synapses; diminished synaptic functions; neuroinflammation; and eventual neurodegeneration. LRP1-KO mice also have memory deficits and movement disorders consistent with compromised dendritic spine/synaptic integrity and synaptic functions. When bred to APP/PS1 amyloid model mice, we also found that both Aβ levels and amyloid plaque loads were increased in LRP1-KO mice.

## Conclusion

Our results clearly demonstrated an important role for apoE receptor LRP1 in brain apoE/lipoprotein metabolism and associated synaptic functions. Because LRP1 levels are reduced in human AD brains, our LRP1-KO mouse model offers an opportunity to study the pathogenic mechanisms of AD. We propose that enhancing apoE and apoE receptor-mediated lipid transport and Aβ clearance in AD brains might be a beneficial strategy to treat AD.

## Acknowledgements

Supprted by: NIH/NIA. Alzheimer's Association, American Health Assistant Foundation.

